# Characterization of the 3D structure of a cultivated land surface and its influence on wheat seedlings growth using Kinect

**DOI:** 10.1038/s41598-017-04392-3

**Published:** 2017-06-20

**Authors:** Tao Liu, Wen Chen, Fujian Li, Wei Wu, Chengming Sun, Jinfeng Ding, Xinkai Zhu, Wenshan Guo

**Affiliations:** Jiangsu Key Laboratory of Crop Genetics and Physiology/Co-Innovation Center for Modern Production Technology of Grain Crops, Yangzhou University, Yangzhou, 225009 China

## Abstract

The quality of wheat emergence has a significant impact on the subsequent growth and development of seedlings. The structure of cultivated land surfaces is an important factor influencing wheat seedlings growth. However, few studies have investigated this. In this study, three-dimensional structural parameters of cultivated land surfaces were collected using 3D imaging equipment, and the effects of different grades of lands on the emergence and growth of wheat were evaluated. The evaluation criteria for the soil blocks was designed according to the ISO-25178-2 standard, and the wheat emergence rate, speed, tillering capacity per plant, dry weight per plant, and final yields of different grades of cultivated land were investigated. The results indicated that the three-dimensional information obtained through the Kinect sensors was reliable. The deviation of measured values from the factual values was trivial. The value of R^2^ was greater than 0.99^**^. The value of RMSE was less than 2 mm. These results describe a method for obtaining three-dimensional structures of land surfaces using 3D cameras and the evaluation of wheat emergence capacity. It can be used as a reference for obtaining three-dimensional cultivated land structures or other similar objects.

## Introduction

Various quality parameters relating to crop emergence have an important impact on the entire growth lifecycle of crops. Quick, even, and complete emergence will shorten the time from sowing to full coverage, improve the temporal and spatial competitiveness of crops, build a good basis for the optimal canopy structure, and maximize high yield potential^1^. Emergence quality can generally be represented by the emergence rate, speed, and the quality of the seedlings. Differences in seedling size as well as uneven seedlings will lead to poor height uniformity in the plants, which is not conducive to the establishment of high-yield group structures. Partial areas of high seedling density will reduce nutrient areas and trigger conflicts among plants, thereby affecting growth potential and reducing productivity. Furthermore, the emergence rate also affects the growth and yield of crops^2^. The emergence rate determines the competitiveness of crops with the surrounding crops, and possibly also influences the completion of flowering and ripening during the growing season^3^. Low quality wheat seedlings are not conducive to the formation of good plant types or high-yield group structures.

Among the multiple factors influencing wheat emergence, the effects of soil surface structures cannot be ignored. The most intuitive surface structure is concerned with the sizes of soil blocks and surface roughness. Soil block size is an important factor in soil structure, affecting the germination and emergence of seeds and ultimately impacting growth and development mainly through soil moisture, temperature, soil air quality, and bulk density^4^. A lack of soil moisture, or an uneven humidity, poor soil moisture content, or insufficient water absorption by seeds will inhibit the germination of seedlings. Excessive water absorption will negatively impact the standard seed breadth, increase the likelihood of fungal attacks, and cause the seeds to rot. Extensive soil preparation, over-large soil blocks, and excessive soil moisture evaporation rates will lead to seeds that are not well situated in the soil and experience difficulties emerging. The size of the soil blocks also affects the soil insulation capacity. Soil temperature is the most obvious and intuitive factor influencing seed emergence^5^. In many cases, temperature fluctuations break seed dormancy and promote their germination^6^. The size of soil blocks may also change the soil aeration capacity. Reactive air, O_2_ and CO_2_, and water vapor affect emergence in different ways. Uneven soil surfaces and over-large soil blocks cause soil aeration and water loss, which is not conducive to seedling emergence. Overly fine soil particles result in under-aerated soil, and when soil CO_2_ concentration is too high, seed germination and emergence are reduced^6^. In addition, soil bulk density affects emergence. Hyatt *et al*.^7^ found that seedling rates decreased when soil compactions changed from low to high, and the bulk density caused by soil blocks varied^7^.

Soil surface structures are an essential aspect of soil quality, and preservation and improvement is key to maintaining soil functioning. Soil structures play an important role in crop emergence and yield^8^. Since soil block size plays an important role in many agricultural phenomena, such as infiltration, runoff, water storage, erosion, heat flux, and evaporation^9^, it is necessary to measure the sizes of soil blocks. Many measurement and evaluation methods have been employed over the years in soil micro-morphology and soil roughness, such as laser scanners^10^, digital photogrammetry^11^, and 3D sensors^12^. However, the implementation of these techniques is subject to various obstacles. Laser scanners are not suitable for a large-scale area analysis and are relatively costly^13^. Although the photogrammetry method is cost effective, it is time consuming for the computation of three-dimensional reconstructions, and has strict requirements for camera calibration. Therefore, the use of three-dimensional sensors to measure sizes of soil blocks has become popular. If the costs for three-dimensional measurements can be made effective, three-dimensional cameras can be used to measure sizes of the soil blocks as well as to evaluate cultivated land surface structures.

In recent years, the advent of fast computer processors has facilitated the development of sensors that are lightweight, simple to operate, and inexpensive, which makes it possible to realize the intelligentization of agricultural machinery. In 2010, Microsoft launched the first Kinect 3D camera, which comprised 6 parts including an infrared transmitter, a color camera, an infrared camera, a microphone array, the base motor, and logic circuits. The infrared emitter and camera provide depth data through launching and receiving infrared rays. The color camera provides RGB data and measures three-dimensional structures through the fusion algorithm. The retail price of Kinect is only $149, which greatly reduces the costs of three-dimensional measurements. Furthermore, Kinect possesses an efficient algorithm and provides real-time access^14^. In order to facilitate the development of three-dimensional application software, Microsoft launched Kinect for Windows ($249) in 2013, which promoted appropriate software development and precipitated its wide use in various fields. Kinect can be used in the monitoring of animals in agriculture^15^, measurement of crop structure parameters^16^, the monitoring of crop growth^17^, and inspection of greenhouse environments^18^. Therefore, if Kinect can be used to measure three-dimensional structures of cultivated land surfaces and identify the effects of different types of three-dimensional structures on wheat emergency, it will inform the accurate cultivation of wheat and provide a reference for the evaluation of cultivated land.

This study utilized Kinect (for Windows) to obtain the three-dimensional information of cultivated land surface structures via three-dimensional reconstruction technologies. The effects of three-dimensional structures on wheat emergence were investigated through the manual construction of different types of cultivated land surface structures, ultimately evaluating the effects of cultivated land surface structures based on the information from Kinect.

## Materials and Methods

### Field experiments and investigation

Field experiments were conducted at a research farm at Yangzhou University, Jiangsu Province, China (32°30′N, 119°25′E, 21 m altitude) during the wheat growing season. In the field experiment, soil samples were screened through different mesh sieves. The soil samples were divided into 10 grades according to the size of the largest soil blocks. The specific grades are shown in Table 1; hydrolysable N: 116.17 mg·kg^−1^, available P: 39.56 mg·kg^−1^, available K: 141.61 mg·kg^−1^, pH: 5.7, 2.6% organic matter content, 43.6% moisture content before sowing, bulk density 1.23 g·cm^−3^. All the grades of soil were from one field. The chemical properties of the soil are the average values of 5 sampling points of the field.

**Table 1:** Test soil grades and compositions.

Grading	Max grain size (mm)	Grain size (mm)					
		<5	5–15	15–50	50–80	80–120	>120
1	4	100%	0%	0%	0%	0%	0%
2	12	58.9%	41.1%	0%	0%	0%	0%
3	22	45.6%	36.7%	17.7%	0%	0%	0%
4	34	38.8%	34.6%	26.6%	0%	0%	0%
5	48	35.3%	33.0%	31.7%	0%	0%	0%
6	64	31.2%	28.6%	26.3%	13.9%	0%	0%
7	82	30.9%	24.2%	20.6%	16.7%	7.6%	0%
8	102	28.3%	21.1%	17.8%	15.6%	17.2%	0%
9	122	25.5%	21.7%	16.7%	13.2%	12.3%	10.6%
10	142	22.3%	20.3%	15.2%	13.0%	14.5%	14.7%

**Figure 1:**
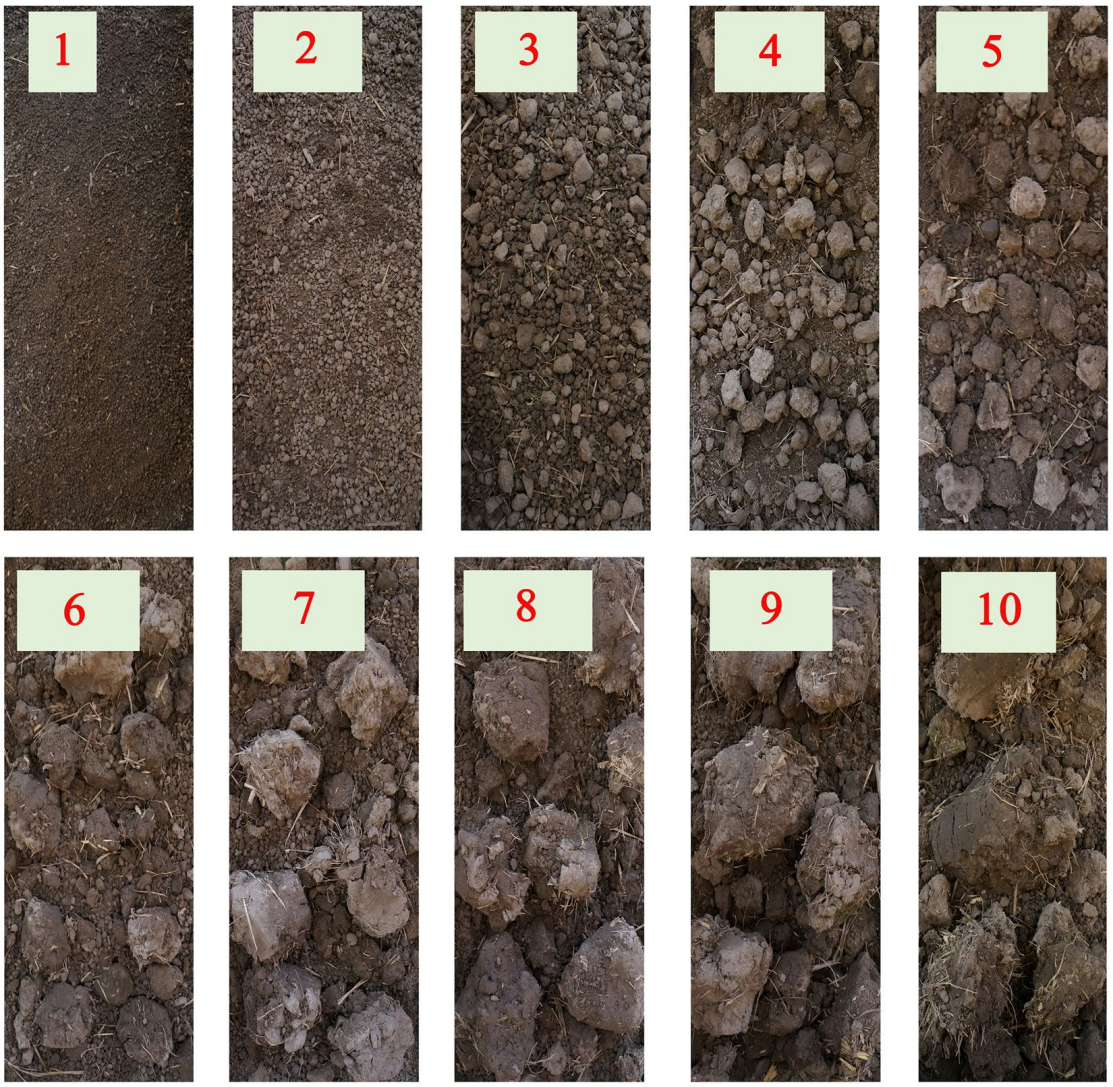
Vertical images of the different grades of soil.

Images of the different grades of soil are presented in Fig. 1.

The experiment was laid out in a two-factor randomized block design with three replicates. The 10 grades of soil and 5 cultivars were selected as the treatments. Plot dimension was 2 m × 3 m and the plots were separated by a 0.3 m wide space. The land was leveled off by tractor. The wheat seeds were broadcast on the flat plot (seeding rate is 250 × 10^4^·ha^−1^). Then, the soil of different grades were lain on the plot respectively. The experimental varieties included 5 samples: Yangmai23, Yangfumai4, Yangmai158, Yangnuomai1, and Xumai33. The emergence rates of the 5 samples respectively were 99.6%, 97.3%, 95.2%, 98.6%, and 98.5%. They were sowed on December 16, 2015 and their daily emergence status was recorded until January 4, 2016. During this time, the lowest recorded temperature was −6 °C and the highest was 16 °C. The tillering status was investigated at the wintering, jointing, and booting stages of wheat growth, and the final yields were calculated. The wheat seedling counting method based on image processing technology which we proposed was used to get seedlings number^19^. The field tiller number per plant survey was performed manually: 20 wheat plants were sampled from each plot (three replication), and the samples were separated according to organs, dried at 105 °C for 30 min, and then at 80 °C to constant weight. The aboveground dry weight was then measured. Spike numbers, grains per spike, and weight per 1000 wheat were investigated from each plot at harvest. Yield was calculated from 1.2 m^2^ area.

The weather conditions during the growth period included an accumulated temperature of 2253 °C, 400 mm precipitation, and 1149 h sunshine duration.

### Image capture

**Figure 2:**
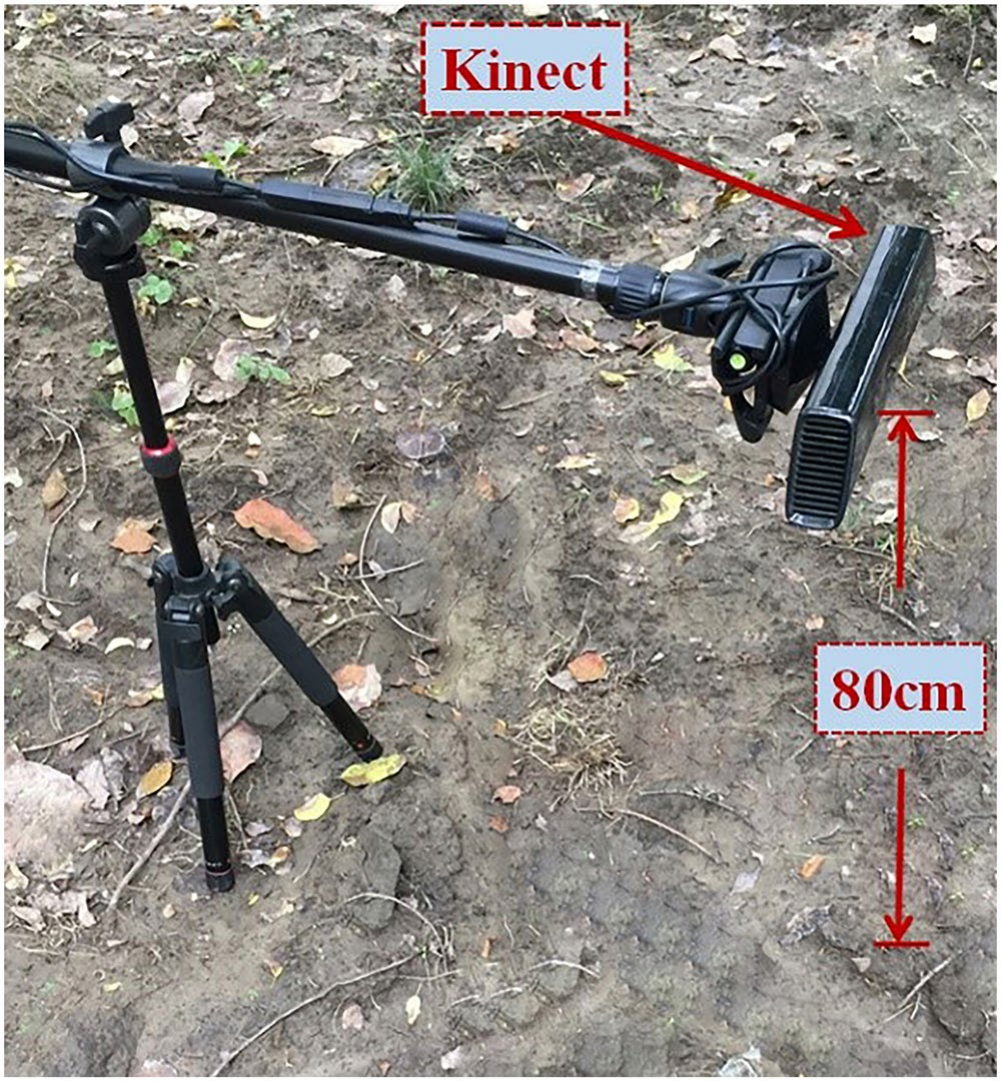
Image capture equipment (Kinect).

Figure 2 shows the image capture devices. To ensure the stability of the image capture, a tripod was used to support the Kinect equipment, and the image sensor was placed to vertically face the cultivated land. The images were captured in the soft daylight and the camera was placed at a height of about 80 cm. Depth images were captured at the same time as RGB images of the cultivated land.

### Image analysis and processing

Microsoft Kinect for Windows includes C language applications, Visual Basic, C#, and an interface Developer Toolkits. The three-dimensional images were processed using these toolkits. Based on the Kinect device characteristics, a value range for the X axis and Y axis of the images were respectively set as: X∈ [0, 640], Y∈[0, 480]. For a point in the three-dimensional coordinate system, P(X, Y, Z), the corresponding point in the two-dimensional coordinate system is p′(*x*′, *y*′); its depth is (*x*′, *y*′); the focal length of the depth camera is: fx_d_ and fy_d_; and the coordinates of the center point of the camera are: c_x_ and c_y_. The relationship between the three-dimensional point (P(X, Y, Z)) and the two-dimensional point (p′(*x*′, *y*′)) is as follows:

1 $$\{\begin{array}{l}X=(x^{\prime} -{{\rm{c}}}_{{\rm{x}}})\times {\rm{depth}}(x^{\prime} ,y^{\prime} )\times \frac{1}{{{\rm{fx}}}_{{\rm{d}}}}\\ Y=(y^{\prime} -{{\rm{c}}}_{{\rm{y}}})\times {\rm{depth}}(x^{\prime} ,y^{\prime} )\times \frac{1}{{{\rm{fy}}}_{{\rm{d}}}}\\ Z={\rm{depth}}(x^{\prime} ,y^{\prime} )\end{array}$$

The image analysis process followed these 3 steps:

1. Firstly, a three-dimensional curved surface chart of the cultivated land was constructed. This study took advantage of the capability of Kinect to capture multi-source information, using RGB images as the measurement criterion for information matching, and converting the two-dimensional space into three-dimensional space using ICP (Iterative Closest Point) and equation (1)^20^, ultimately achieving a three-dimensional image of the cultivated land using the Delaunay triangulation algorithm^21, 22^. The resulting three-dimensional image is defined as: G(*r*), which consists of 2 parts: flat land (G(*r*)_basal_), and bulgy soil blocks (G(*r*)_block_). Their relationship is as follows:
  2 $${\rm{G}}(r)={\rm{G}}{(r)}_{{\rm{basal}}}+{\rm{G}}{(r)}_{{\rm{block}}}$$
2. Secondly, soil blocks were extracted and their heights were measured from the depth image, which can be viewed as a topographic map. In this study, soil blocks were extracted from the three-dimensional image using the Gaussian low pass filter (equations (3), σ = 0.05)^23^. After the filtering, G(*r*)_basal_ and G(*r*)_block_ were respectively defined as 0 and 1 according to the threshold value. The figurations of soil blocks were extracted. The sizes and heights of the soil blocks were calculated based on their depths.
  3 $${\nabla }^{2}{\rm{G}}(p)=\frac{1}{2{{\rm{\pi }}{\rm{\sigma }}}^{2}}(2-\frac{{x}^{2}+{y}^{2}}{{{\rm{\sigma }}}^{2}}){e}^{(-{x}^{2}+\frac{{y}^{2}}{2{{\rm{\sigma }}}^{2}})}$$
3. Lastly, the three-dimensional characteristic parameters of the soil blocks were calculated. According to the definitions of height parameters specified in ISO-25178-2(2012), 4 parameters, S_a_, S_q_, S_sk_, and S_ku_, were selected as indicators to evaluate the three-dimensional structure of the cultivated land^24^.

4 $${{\rm{S}}}_{{\rm{a}}}=\frac{1}{A}{\iint }_{A}|{\rm{z}}(x,y)|{\rm{dxdy}}$$

5 $${{\rm{S}}}_{{\rm{q}}}=\sqrt{\frac{1}{A}{\iint }_{A}{{\rm{z}}}^{2}(x,y){\rm{dxdy}}}$$

6 $${{\rm{S}}}_{{\rm{sk}}}=\frac{1}{{{{\rm{S}}}_{{\rm{q}}}}^{3}}[\frac{1}{A}{\iint }_{A}{{\rm{z}}}^{3}(x,y){\rm{dxdy}}]$$

7 $${{\rm{S}}}_{{\rm{ku}}}=\frac{1}{{{{\rm{S}}}_{{\rm{q}}}}^{4}}[\frac{1}{A}{\iint }_{A}{{\rm{z}}}^{4}(x,y){\rm{dxdy}}]$$

These 4 indicators can be used not only to evaluate the sizes of the soil blocks on the land, but also to evaluate the roughness, changing scales of the land surface and measure the quality of the land from multiple perspectives. The pin meter method was used to get these 4 indicators. These 4 indicators got by the pin meter method were selected as the manual measurement result. The surface characteristics of different plots are shown in Table 2.

**Table 2:** Characteristics of different grades of cultivated land.

Grading	Sa	Sq	Ssk	Sku
1	1.28	1.46	1.26	1.66
2	4.00	4.72	1.38	2.08
3	6.94	9.96	2.16	5.66
4	9.80	12.85	1.65	3.04
5	10.93	14.90	1.70	3.22
6	17.89	27.13	1.98	4.38
7	31.83	45.63	1.74	3.31
8	36.98	52.09	1.58	2.67
9	45.29	63.51	1.64	2.87
10	52.23	73.73	1.63	2.82

Six sets of data were obtained from each treatment. The data were divided into two data sets, which were then used for building the method (150 observations) and validating the method (150 observations), respectively. The performance of the method was evaluated by using coefficients of determination of models (R^2^), the root mean square error in prediction (RMSE), and the relative error in prediction (REP). R^2^ and RMSE were used to evaluate the stability of the model and the average deviation between the measured values and the true values. REP was used to measure the accuracy of the model with respect to prediction.

## Results and Analysis

### Three-dimensional construction of cultivated land

**Figure 3:**
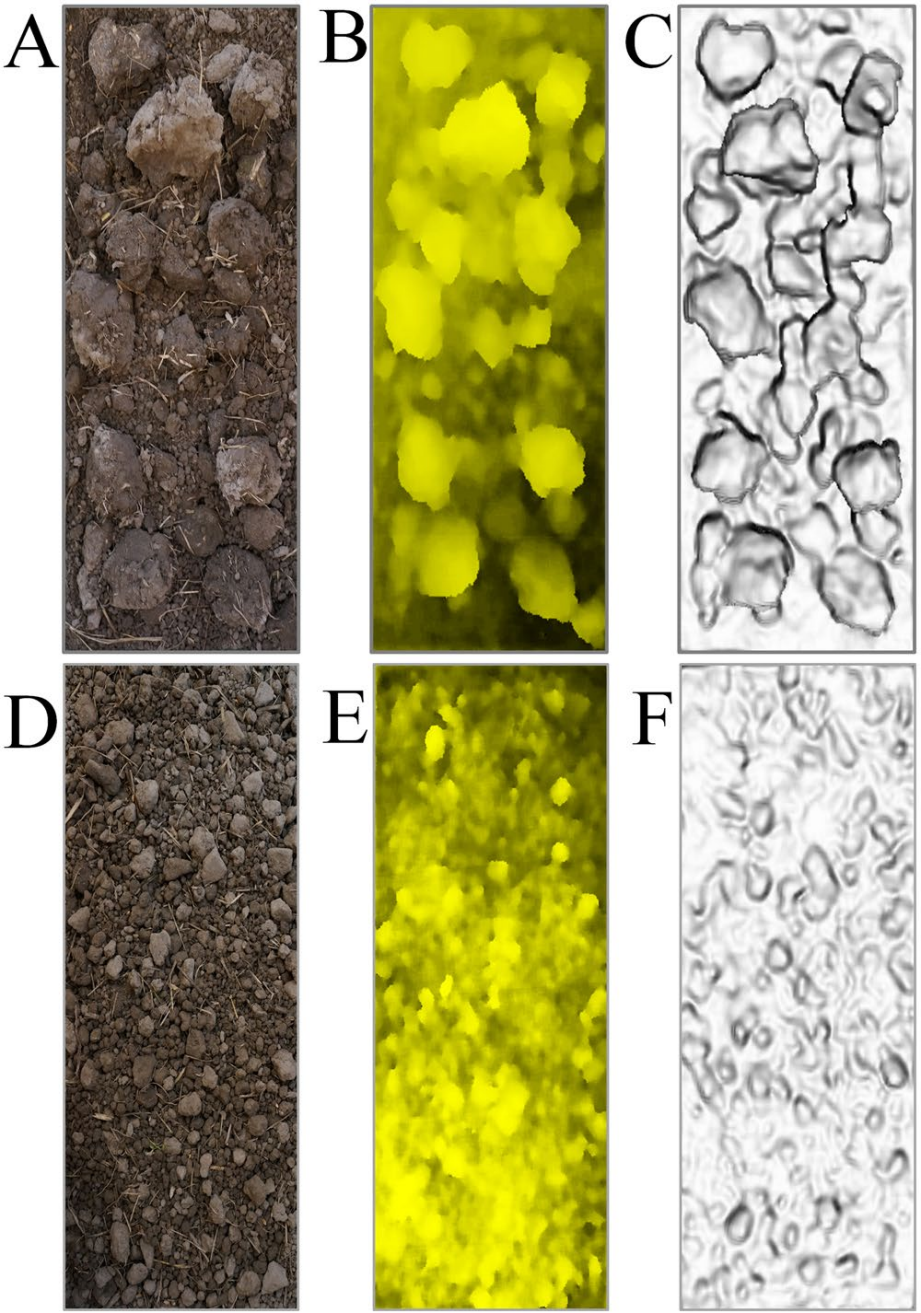
Preprocessing. (**A**) Color image of grade 6; (**B**) Depth image of grade 6; (**C**) Three-dimensional image of grade 6; (**D**) Color image of grade 3; (**E**) Depth image of grade 3; (**F**) Three-dimensional image of grade 3.

Figure 3 shows the original image, depth image, and three-dimensional image of one of the cultivated land samples. As indicated in Fig. 3, the three-dimensional information of soil blocks in the land was well constructed using Kinect equipment and Equation (1) mentioned in Section 1.3.

**Figure 4:**
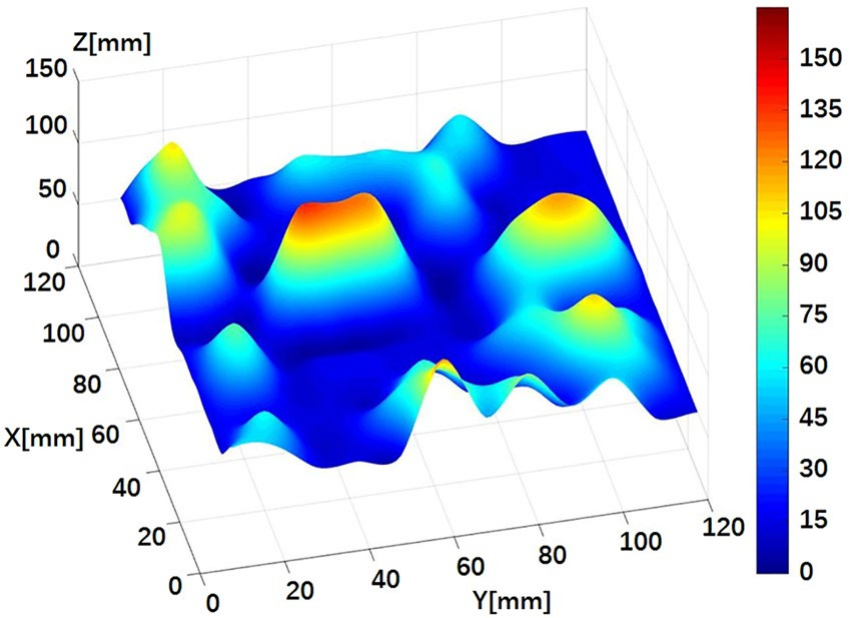
Three-dimensional space image of a sample of cultivated land.

The sizes of the soil blocks were easily captured from the constructed three-dimensional image of the land in the three-dimensional coordinate system (Fig. 4). As indicated in Fig. 4, the spatial coordinates were converted into the actual sizes. The lengths and widths of the soil blocks were calculated using the X and Y values, while the heights were calculated using the value of Z.

### Characteristics of the different grades of cultivated land

The differences among the values of Sa and Sq were significant, and could be used to evaluate soil grades. No significant differences existed among the values of Ssk and Sku. A small difference existed between grade 1 and grade 2 lands with respect to the values of Ssk and Sku.

**Figure 5:**
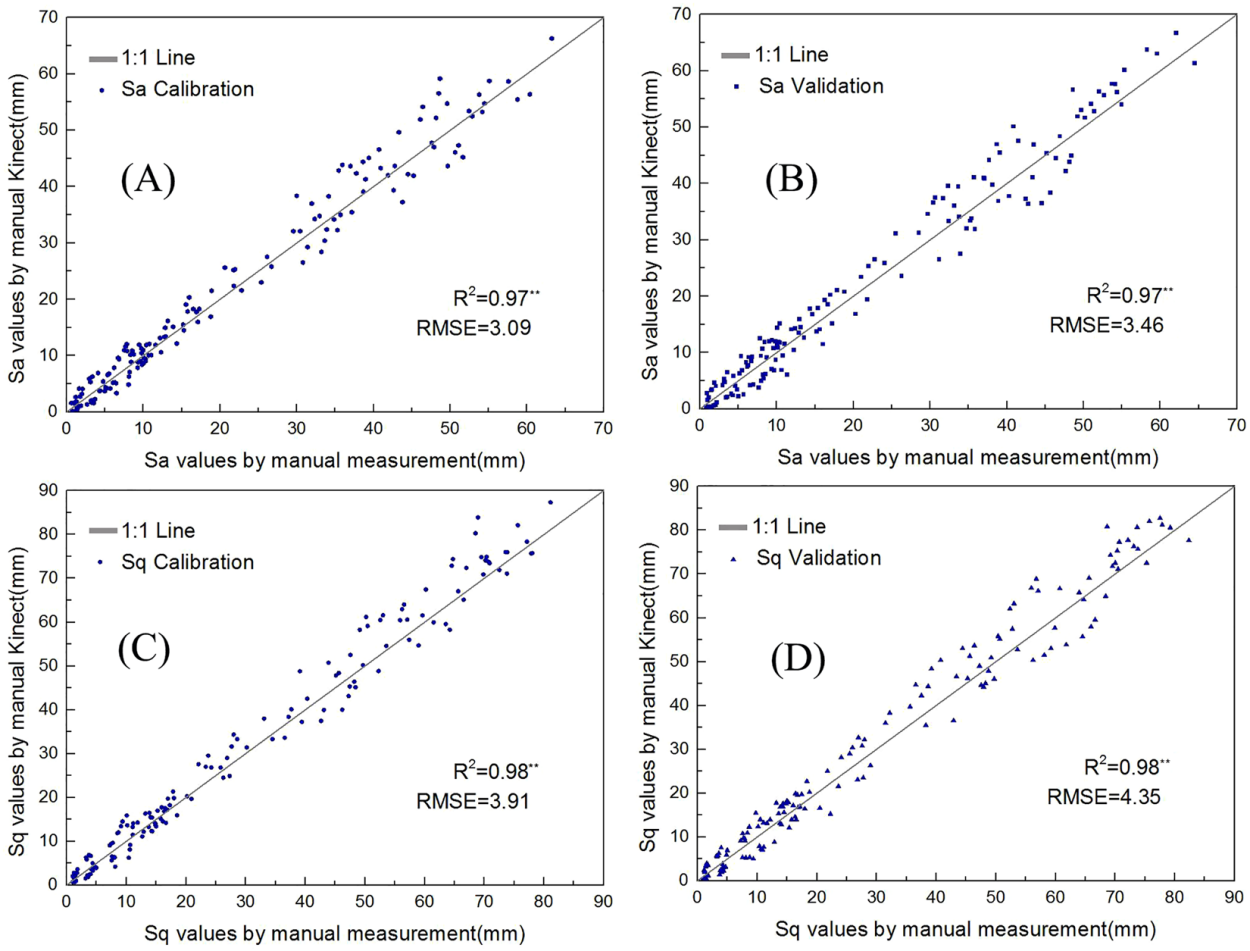
Comparison between the manual and Kinect measured value of Sa Calibration (**A**), Sa Validation (**B**), Sq Calibration (**C**), Sq Validation (**D**).

Figure 5 is a 1:1 line graph for the values of Sa and Sq measured manually and by using Kinect. As shown in Fig. 5, the values measured in these 2 approaches were closely matched. The correlation coefficient (R^2^) of the 2 values of Sa were 0.97^**^ (Calibration) and 0.97^**^ (Validation), and the RMSE were 3.09 (Calibration) and 3.46 (Validation). The correlation coefficient (R^2^) of the 2 values of Sq were 0.98^**^ (Calibration) and 0.98^**^ (Validation), and the RMSE were 3.92 (Calibration) and 4.35 (Validation). The above results indicate that the characterisitic values measured by Kinect are accurate, providing a basis for the later grade classification of cultivated land.

Table 3 is the statistical results of different grades for the values of Sa and Sq measured manually and by using Kinect. For the calibration data set, R^2^ was 0.65–0.88, RMSE was 1.92–5.45, REP was 2.87–50.48%. For the validation data set, R^2^ was 0.57–0.84, RMSE was 2.11–5.96, REP was 9.66–56.25%. This method performed better on high grades of soil than the low grades for the reason of depth resolution of Kinect.

**Table 3:** Statistics for evaluating the performance of proposed method at different grades of soil in calibration and validation datasets

	parameters	R^2^	RMSE	REP
Calibration	Sa_1–3_	0.72	2.25	45.96
	Sa_4–6_	0.86	2.83	15.39
	Sa_7–10_	0.89	5.45	8.15
	Sq_1–3_	0.66	1.92	50.48
	Sq_4–6_	0.84	2.09	15.56
	Sq_7–10_	0.84	4.30	2.87
Validation	Sa_1–3_	0.68	2.28	56.98
	Sa_4–6_	0.79	3.28	18.61
	Sa_7–10_	0.83	5.96	9.66
	Sq_1–3_	0.57	2.11	59.25
	Sq_4–6_	0.71	2.72	21.81
	Sq_7–10_	0.83	4.63	10.47

_1–10_ are different grades of soil.

**Figure 6:**
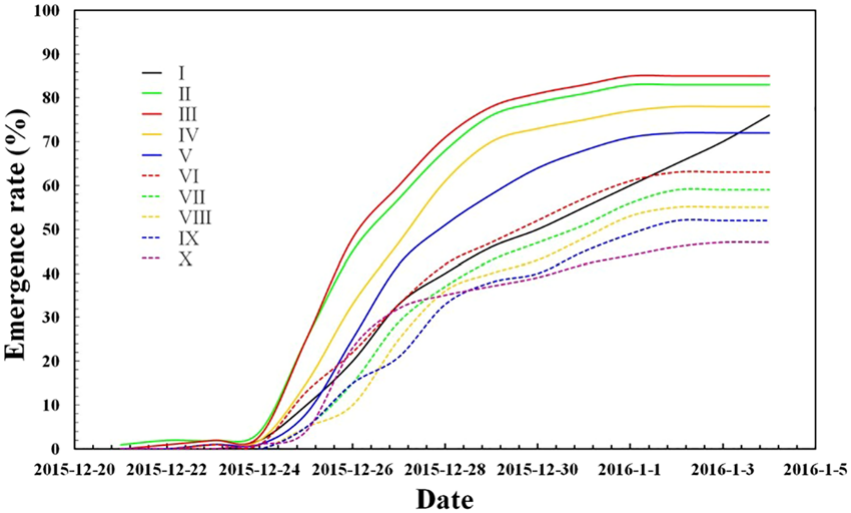
Emergence process of different grades of cultivated land.

Different grades of cultivated land had a significant impact on wheat emergence. Figure 6 shows the emergence process of 5 wheat varieties in different grades of cultivated land. Lands of grades II, III, and IV possessed high emergence rates, as well as fast emergence speeds. Seeds began to emerge 7 days following sowing, with the emergence rate reaching a maximum at 15 days. The seeds were slow to emerge on the flat surface of I-grade land. However, the later emergence rate on this surface was over 70% and reached a maximum 20 days following sowing.

**Figure 7:**
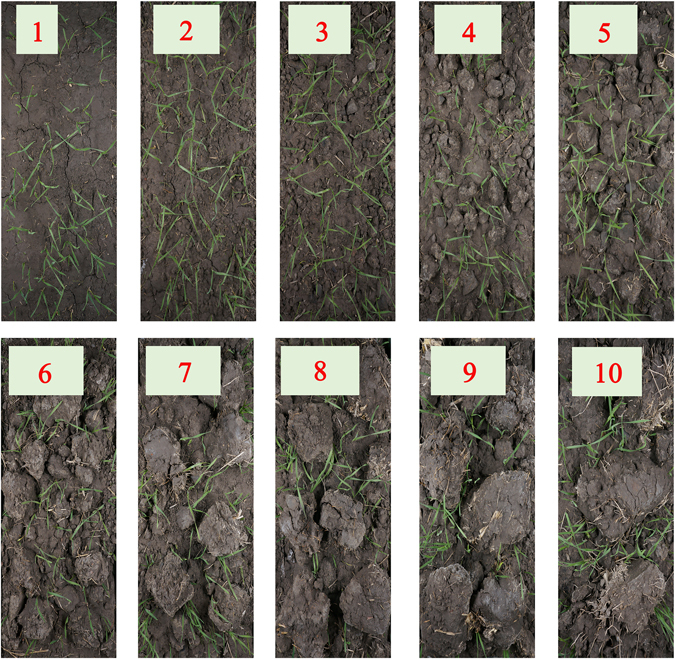
Emergence efficiencies of different grades of cultivated land.

Figure 7 shows the emergence status across the different grades of land. Larger soil blocks reduced the growth space, thereby restricting seedling growth. Similarly, over-fine soil particles were not conductive to emergence.

The surface characteristics of cultivated land not only affected the emergence rate, but also significantly impacted subsequent growth. As shown in Table 4, the number of tillers per plant and the dry weight per plant in the wintering period were different across the different grades of cultivated land. These differences were extremely significant in some populations and impacted the final yields.

**Table 4:** Wheat seedling characteristics of different grades of cultivated land.

	Emergence rate	TN (kg 10^4^ Plants)	DR (kg ha^−1^)	Yield (kg ha^−1^)
1	76.35%	3.71a	2.59a	583.75a
2	83.26%	3.56a	2.46a	596.21a
3	85.17%	3.37ab	2.13ab	584.95a
4	78.61%	2.93b	1.86b	577.62a
5	72.39%	2.86b	1.89b	550.62ab
6	63.25%	2.25bc	1.83b	542.76b
7	59.49%	2.13c	1.63c	531.2b
8	55.06%	1.87d	1.55c	500.92c
9	52.82%	1.68d	1.57c	490.21c
10	47.53%	1.57d	1.51c	480.63c

TN is tiller number per plant at the beginning of wintering stage, and DR is dry weight per 10^4^ plants. Within a column, means followed by the same letter are not significantly different at the 0.05 level of probability by students t test.

## Discussion

### Three-dimensional information capture of cultivated land

The use of two-dimensional images in the intelligent measurement or evaluation of agriculture has become very popular, including the measurement of the number of fruits, the monitoring of pests and disease, and the evaluation of crop group growth^25–27^. Compared to three-dimensional information capture, measurement and evaluation using two-dimensional spatial data are relatively simple, and the applied algorithm is also simple. However, it is not feasible to use two-dimensional images to effectively describe the length, width, and height of cultivated land, which is a three-dimensional object. In this case, three-dimensional measurement technologies must be applied. Traditional three-dimensional measurement techniques include laser scanning and structured light scanning^28, 29^. These techniques have high three-dimensional measurement accuracy. However, their applications are complex and they are expensive. They are more often utilized for the measurement of precision devices in industrial production, and it is not realistic to use them in agriculture in terms of both cost and practicality.

The accuracy does not have to be at the millimeter level for the evaluation of the three-dimensional structure of cultivated land; the accurate description of fluctuations in the land surface is sufficient. A large number of experiments have demonstrated that Kinect is highly accurate for the measurement of soil blocks, with an error that can be controlled within 1 cm, which is sufficient for the “rough” measurement of cultivated land^6, 23, 30, 31^. More importantly, Kinect is cost effective, which makes its extensive application possible.

### Evaluation of cultivated land surface structure

The main purpose of cultivated land evaluation is to determine whether the land is suitable for crop growth. Previous studies have focused more on the influence of nutrients, organic matter, water, and air contained present in an area on crop emergence and subsequent growth. However, the effects of land surface structures have not been explored. This stems from the difficulties in measuring the three-dimensional surface structure of land, as well as a lack of supported equipment. In this study, we were able to capture accurate sizes of land surface soil blocks (length, width, height). This was derived from the 2 indicators, Sa and Sq, which proved ideal for the measurement of soil block roughness and size based on the soil surface structure evaluation standard specified in ISO-25178-2 (2012) and the experimental results of this study (Table 2). It was also demonstrated that the 2 parameters acquired by Kinect are reliable and can be used for soil evaluation.

### Emergence evaluation

According to the results of emergence rate and the later growth in 10 grades of cultivated land, land with more reasonable structures (grades 3, 2, or 4) was conductive to the emergence and subsequent growth of seedlings. The grade 1 land containing overly fine particles exhibited slower emergence speeds and lower rates. The major factors limiting emergence included poor air permeability and high CO_2_ concentration in the soil^32^. Land with rougher surfaces and larger soil blocks (grades 6, 7, 8, 9, or 10) exhibited lower emergence rates. The tiller per plant capacity and dry weight per plant decreased with the increase of the 2 indicators, Sa and Sq, ultimately resulting in poor yields. The primary explanation was that the larger soil blocks inhibited absorption of nutrients and water by the seeds. Alternatively, large soil blocks suppressed seed germination by physically hampering emergence. Spindly or weak seedlings occurred more frequently in the presence of large soil blocks. When planting wheat in grade 1 land, additional soil tillage, less watering, and an increase in soil permeability should help to improve the emergence rate. For land of grade 5 or 6, simple soil crushing will be helpful. Land of grade 7, 8, 9, or 10 would require several applications of crushing by means of a tractor to facilitate seedling emergence and subsequent growth.

### Problems and prospects

This study designed a descriptive method for cultivated land surface structures based on Kinect three-dimensional cameras, with high description accuracy. The effects of different land structures on wheat emergence and subsequent growth were also identified. Due to limited time, an in-depth investigation into several characteristics pertaining to different grades of land including porosity, water, air, heat in soil, and biological and chemical characteristics, was not achieved. This information could offer further explanations for the differences in wheat emergence rates. In addition, sunlight also exerts a certain impact on the Kinect image capturing equipment, in that strong light easily creates holes in the image construction. Therefore, in this study, images were captured during the morning or at dusk in the presence of soft light.

## Conclusions

This paper introduces an evaluation method for cultivated land surface structures using inexpensive Kinect three-dimensional imaging equipment. The acquired data is accurate and the operation is simple. Additionally, the effects of different surface structures on wheat emergence were identified, and the effects of the three-dimensional surface structures on wheat emergence based on Kinect were evaluated.

## References

[1] Soltani A, Robertson MJ, Torabi B, Yousefi-Daz M, Sarparast R (2006). Modelling seedling emergence in chickpea as influenced by temperature and sowing depth. Agricultural & Forest Meteorology..

[2] Gan Y, Stobbe EH, Moes J (1992). Relative date of wheat seedling emergence and its impact on grain yield. Cropence.

[3] Wang H (2009). Predicting the time to 50% seedling emergence in wheat using a Beta Model. Njas Wageningen Journal of Life Sciences.

[4] Sandri R, Anken T, Hilfiker T, Sartori L, Bollhalder H (1998). Comparison of methods for determining cloddiness in seedbed preparation. Soil Till Res..

[5] Kamkar B, Ahmadi M, Soltani A, Zeinali E (2008). Evaluating Non-Linear regression models to describe response of wheat emergence rate to temperature. Configuring Juniper Networks Netscreen & Ssg Firewalls.

[6] Forcella F, Arnold RLB, Sanchez R, Ghersa CM (2000). Modeling seedling emergence. Field Crop Res.

[7] Hyatt J, Wendroth O, Egli DB, Tekrony DM (2007). Soil compaction and soybean seedling emergence. Crop Sci.

[8] Mueller L (2009). Visual assessment of soil structure: Evaluation of methodologies on sites in Canada, China and Germany: Part I: Comparing visual methods and linking them with soil physical data and grain yield of cereals. Soil and Tillage Research.

[9] Kamphorst EC (2000). Predicting depressional storage from soil surface roughness. Soil Sci Soc am J.

[10] Darboux F, Huang C (2003). An instantaneous-profile laser scanner to measure soil surface microtopography. Soil Sci Soc am J.

[11] Aguilar MA, Aguilar FJ, Negreiros J (2009). Off-the-shelf laser scanning and close-range digital photogrammetry for measuring agricultural soils microrelief. BIOSYST ENG..

[12] Marinello, F., Pezzuolo, A., Gasparini, F. & Sartori, L. Three-dimensional sensor for dynamic characterization of soil microrelief (John V.) 71–78 (Wageningen Academic Publishers, 2013).

[13] Draelos, M., Deshpande, N. & Grant, E. The Kinect up close: Adaptations for short-range imaging. *IEEE International Conference on Multisensor Fusion and Integration for Intelligent Systems*. 251-256 (2012).

[14] Stommel M, Beetz M, Xu W (2014). Inpainting of missing values in the Kinect sensor’s depth maps based on background estimates. Sensors Journal IEEE.

[15] Kongsro J (2014). Estimation of Pig Weight Using a Microsoft Kinect Prototype Imaging System. Computers & Electronics in Agriculture.

[16] Chen, Y., Zhang, W., Yan, K., Li, X. & Zhou, G. Extracting corn geometric structural parameters using Kinect. *Nrnaonal Jornal of Rmo Nng*. 6673–6676 (2012).

[17] Fu, D., Xu, L., Li, D. & Xin, L. Automatic detection and segmentation of stems of potted tomato plant using Kinect. *International Conference on Digital Image Processing*. doi:10.1117/12.2064003 (2014).

[18] Nissimov S, Goldberger J, Alchanatis V (2015). Obstacle detection in a greenhouse environment using the Kinect sensor. Computers & Electronics in Agriculture.

[19] Liu T (2016). Automated image processing for counting seedlings in a wheat field. Precis Agric..

[20] Zhang C, Du S, Liu J, Xue J (2016). Robust 3D point set registration using iterative closest point algorithm with bounded rotation angle. Signal Process..

[21] Liu Y, Lo SH, Guan Z, Zhang H (2014). Boundary recovery for 3D delaunay triangulation. Finite Elem Anal Des.

[22] Dou J, Li J (2014). Image matching based local delaunay triangulation and affine invariant geometric constraint. Optik - International Journal for Light and Electron Optics..

[23] Chávez GM, Sarocchi D, Santana EA, Borselli L, Rodríguez-Sedano LA (2014). Using Kinect to analyze pebble to block-sized clasts in sedimentology. Comput Geosci-UK..

[24] Marinello F, Pezzuolo A, Gasparini F, Arvidsson J, Sartori L (2015). Application of the Kinect sensor for dynamic soil surface characterization. Precis Agric..

[25] Liu T (2016). Detection of aphids in wheat fields using a computer vision technique. Biosyst Eng..

[26] Lee K, Lee B (2013). Estimation of ice growth and nitrogen nutrition status using color digital camera image analysis. Eur J Agron..

[27] Appoloni CR, Pottker WE (2005). Non-destructive porosity profile measurement of amorphous materials by Gamma-Ray transmission. Applied Radiation & Isotopes Including Data Instrumentation & Methods for Use in Agriculture Industry & Medicine..

[28] Nurunnabi A, West G, Belton D (2015). Outlier detection and robust normal-curvature estimation in mobile laser scanning 3D point cloud data. Pattern Recogn..

[29] Davis, J. & Chen, X. A laser range scanner designed for minimum calibration complexity. *Third International Conference on on 3D Digital Imaging & Modeling*, 2001:91–98.

[30] Kongsro J (2014). Estimation of pig weight using a Microsoft Kinect prototype imaging system. Comput Electron Agr..

[31] Mankoff KD, Russo TA (2013). The Kinect: A low-cost, high-resolution, short-range 3D camera. Earth Surface Processes & Landforms..

[32] Alm DM, Wax LM (1993). An index model for predicting seed germination and emergence rates. Weed Technol..

